# HCV Core Protein-Induced Down-Regulation of microRNA-152 Promoted Aberrant Proliferation by Regulating Wnt1 in HepG2 Cells

**DOI:** 10.1371/journal.pone.0081730

**Published:** 2014-01-09

**Authors:** Shifeng Huang, Yan Xie, Ping Yang, Pu Chen, Liping Zhang

**Affiliations:** Department of Laboratory Medicine, the First Affiliated Hospital of Chongqing Medical University, Chongqing, China; French National Center for Scientific Research - Institut de biologie moléculaire et cellulaire, France

## Abstract

**Background:**

Hepatitis C virus (HCV) has been reported to regulate cellular microRNAs (miRNAs). The HCV core protein is considered to be a potential oncoprotein in HCV-related hepatocellular carcinoma (HCV-HCC), but HCV core-regulated miRNAs are largely unknown. Our preliminary experiments revealed significant down-regulation of microRNA-152 (miR-152) by HCV core protein in HepG2 cells. Through target gene prediction softwares, Wnt1 was predicted to be a potential target of miR-152. The present study was initiated to investigate whether miR-152 is aberrantly regulated by the HCV core protein, and involved in the regulation of the aberrant proliferation of HCV-HCC cells.

**Methods:**

MiR-152 levels were examined by stem-loop real-time RT-PCR (SLqRT-PCR). Cell proliferation was analyzed by MTT and colony formation assay. Cell cycle analysis was performed by flow cytometry. Luciferase reporter assay was conducted to confirm miRNA-target association. Wnt1 expression was determined by real-time qPCR and Western blotting.

**Results:**

HCV core protein significantly suppressed miR-152 expression, and led to significant Wnt1 up-regulation with a concomitant aberrantly promoted proliferation. Moreover, we validated that miR-152 inhibition promoted, while miR-152 mimics inhibited cell proliferation. Using, qRT–PCR and western blot, Wnt1 was demonstrated to be regulated by miR-152. Luciferase activity assay showed that while miR-152 mimics significantly reduced the luciferase activity by 83.76% (P<0.0001), miR-152 inhibitor showed no effect on luciferase reporter. Most notably, salvage expression of miR-152 after Ad-HCV core infection for 24 h almost totally reversed the proliferation-promoting effect of the HCV core protein, and meanwhile, reduced the expression of both Wnt1 mRNA and protein to basal levels.

**Conclusion:**

These findings provide important evidence that the reduced miR-152 expression by HCV core protein can indirectly lose an inhibitory effect on Wnt1, which might, at least partially lead to cell proliferation of liver cancer cells. MiR-152 may have a therapeutic potential to suppress liver cancer proliferation.

## Introduction

Hepatocellular carcinoma (HCC) is the main type of liver cancer and the third most common cause of cancer mortality world-wide [1]. Infection with hepatitis C virus (HCV) is one of the major risk factors for the development of HCC [2]. HCV-related HCC arises as a result of many genetic and epigenetic alterations. It was repeatedly reported that epigenetic factors such as DNA methylation-associated gene silencing [3], [4] and microRNAs (miRNAs) deregulations [5] significantly impact the course of HCV-related HCC development.

MiRNAs are noncoding RNAs that have been highly conserved during evolution and have emerged recently as potent regulators of gene expression, proliferation and cancer [6]–[8]. Importantly, miRNAs are emerging as important players in liver health and disease, and involvements of miRNAs were demonstrated in hepatocyte apoptosis [9], liver fibrosis [10] and hepatocarcinogenesis [8]. What's more, miRNAs deregulations have been very recently demonstrated to recapitulate hepatic oncogenesis in various animal models [11], [12], [13].

Ever increasing pieces of evidence have been indicating that the presence of HCV in HCC significantly associates with aberrant miRNAs expressions [14], [15]. More notably, several recent studies have reported a strong correlation between HCV infection and deregulation of many miRNAs, such as miR-155 [14], miR-491 [15], miR-26b [16], miR-193b [5] and miR-124 [17], [18], suggesting that a dysfunction of oncomirs or tumor suppressive miRNAs may be associated with HCV-related hepatocarcinogenesis. Actually, a very recent study showed that HCV-induced miR-155 expression promoted hepatocyte proliferation and tumorigenesis by activating Wnt signaling [14].

The HCV core protein is considered to be a potential oncoprotein in HCV-related hepatocellular carcinoma (HCV-HCC), but HCV core-regulated microRNAs are largely unknown.

MicroRNA-152 (miR-152) has been reported to be substantially down-regulated in some solid tumors, including breast cancer [19], endometrial cancer [20], ovarian cancer [21], cholangiocarcinoma [22] and HBV-related HCC [23]. MiR-152 was previously shown to be down-regulated by hepatitis B virus (HBV) X protein and functions as a tumor suppressive miRNA by targeting the dnmt1 gene in HBV-associated HCC. Moreover, ectopic expression of miR-152 in HCC cells was shown to have inhibited cell growth [23]. These findings support a tumor-suppressive role of miR-152 in the epigenetic aberration of HBV-related HCC and the potential development of miR-152-based strategy for the treatment of HBV-related HCC. Additionally, miR-152 has been reported to be an tumor suppressive miRNA that is silenced by DNA hypermethylation in endometrial cancer, and restoration of miR-152 expression in endometrial cancer cell lines was sufficient to inhibit tumor cell growth *in vitro* and *in vivo*, with *E2F3*, *MET*, and *Rictor* being its novel candidate targets [20]. More recently, Zhou and colleagues [21] reported that down-regulated expression of miR-152 in ovarian cancer is related to deregulated cell proliferation, and miR-152 may be a novel biomarker for early detection and/or a therapeutic target of ovarian cancer. These data all suggest a tumor suppressive function of miR-152. However, its expression and pathological function in HCV-related HCC is still unknown. In our preliminary experiments, HepG2 cells infected with Ad-HCV core (HepG2-HCV) or the Ad-EGFP control (HepG2-control) were profiled for differential miRNA expression using the Affymetrix miRNA array platform. miRNA microarray analysis of HepG2-HCV and HepG2-control cells showed 6 differentially expressed miRNAs between these cells with a fold-difference of greater than or equal to 1.5 and p-values≤0.05. Among which miR-152 was the only one validated by qRT-PCR to be significantly down-regulated by the over-expression of the HCV core protein (unpublished data). Through target gene prediction softwares, the proliferation-promoting oncogene, Wnt1, was predicted to a potential target of miR-152.

Herein, we report that down-regulation of miR-152 by HCV core protein in HCC is important in the acquisition of a malignant proliferation phenotype. Ectopic over-expression of miR-152 in HepG2 cells is sufficient to inhibit cell proliferation, while on the opposite, miR-152 inhibition promoted cell proliferation. More importantly, we provide the first evidence that miR-152 can modulate Wnt1 expression, but possibly through an unknown target of miR-152 which in turn leads to the regulation of Wnt1. Collectively, our results provide an explanation for the malignant proliferative nature of HCV core protein induced-HCC, linking this aggressive nature mechanistically to the HCV core-miR-152-Wnt1 pathway.

## Materials and Methods

### Cell culture and transfection

HepG2 cells were purchased from ATCC and cultured in Dulbecco's modified Eagle's medium (DMEM, Hyclone) supplemented with 10% (v/v) FCS, 2 mmol/L glutamine, 100 units/mL penicillin, and 100 µg/mL streptomycin at 37°C in a humidified chamber. Transfections were performed with a HiPerFect Transfection Reagent kit (Qiagen) according to the manufacturer's instructions. For MTT, cell cycle profile analysis and colony formation assay, double-stranded miR-152 mimics, single-stranded miR-152 inhibitor, or their relative negative control RNA (Qiagen) at a final concentration of 5 nM was introduced into cells.

### Construction of the Ad-HCV core adenovirus and the infection of HepG2 cells

Both the Ad-HCV core adenovirus and the control Ad-EGFP adenovirus were constructed with the Ad-easy system. HepG2 cells were plated into a 6-well plate in 1000 µl of complete medium at a density of 3×10^5^ cells per well and incubated overnight until 50–80% confluent. The Ad-HCV core adenovirus and the control Ad-EGFP adenovirus were respectively infected into HepG2 cells at an MOI = 50. The infection efficiency was evaluated by observation of EGFP expression under fluorescent microscope at 48 h after infection. Cells were harvested at 48 h following infection and used to prepare for total RNA, protein, flow cytometry and colony formation assay.

### miRNAs isolation and Stem-loop real-time RT-PCR

Total RNA enriched with miRNAs was isolated from HepG2 cells using the mirVana PARIS miRNA isolation kit (Ambion) according to the manufacturer's instructions. To determine the expression level of miR-152, stem-loop real-time RT-PCR (SLqRT-PCR) was performed. MiRNAs were quantified by using TaqMan miRNA quantitative reverse transcriptase-polymerase chain reaction (qRT–PCR) assay according to the protocol of the manufacturer (Applied BioSystems, Inc.). Briefly, total RNA (10 ng) was used for first-strand cDNA synthesis using miRNA-152-specific, stem-loop primer, or U6 stem-loop primer, a control endogenous miRNA (Applied Biosystems, Foster City, CA), followed by real-time PCR amplification with gene-specific forward primer and a reverse primer along with a probe, in an ABI Prizm 7500 PCR machine. The relative miR-152 expression was calculated from three different experiments. All reactions were run in triplicate, and results were normalized to those for U6 RNA. Relative miR-152 production was determined with the ΔCt method and reported as 2^−ΔΔCt^, where Ct is the threshold cycle, ΔCt = Ct_miR-152_-Ct_U6_, ΔΔCt = ΔCt_Ad-Ctrl/Ad-Hcv_-ΔCt_Mock_. After normalization to endogenous U6 expression, the expression of miR-152 in each group was expressed as fold changes in comparison to the levels observed in mock-infected HepG2 cells.

### RNA isolation and quantitative reverse transcription-polymerase chain reaction (qRT-PCR) analysis for Wnt1 mRNA

Total RNA was extracted by SV Total RNA Isolation system (Promega, USA). One microgram of total RNA was reverse-transcribed using the PrimeScript® RT reagent Kit (TaKaRa Biotechnology, Dalian, China). Real-time quantitative PCR (q-PCR) was performed with a standard SYBR-Green PCR kit protocol on a ABI 7500 Real-time PCR System (Applied Biosystems, USA). The primer sequences used were as follows: for Wnt1, 5′-CCCTAACCGGTGCGCCCTGGTGCC-3′ (forward) and 5′-AGCGCCCAGAGCCCCATGGCCTGC-3′ (reverse); for GAPDH, 5′-ACCCAGAAGACTGTGGATGG-3′ (forward) and 5′-TCTAGACGGCAGGTCAGGTC-3′ (reverse). All measurements were performed in triplicate and the relative Wnt1 levels were calculated using the Comparative Ct Method Relative Wnt1 mRNA production was determined with the ΔCt method and reported as 2^−ΔΔCt^, where Ct is the threshold cycle, ΔCt = Ct_Wnt1_-Ct_GAPDH_, ΔΔCt = ΔCt_Ad-Ctrl/Ad-Hcv_-ΔCt_Mock_. After normalization to endogenous GAPDH expression, the expression of Wnt1 mRNA in each group was expressed as fold changes in comparison to the levels observed in mock-infected HepG2 cells.

### Western blot Analysis

M-PER Mammalian Protein Extraction Reagent (cell signaling) was employed to lyze cells. Protein were resolved on 10% SDS-PAGE gels and transferred to PVDF membranes. Membranes were blocked with 5% BSA in TBS and 0.1% Tween-20, and incubated with primary rabbit polyclonal anti-Wnt1 antibody (Abcam, Cambridge, UK) or mouse monoclonal anti-Flag (a flag tag was added into the Ad-HCV core vector) antibody (Cell signaling Technology, Inc.) and horseradish peroxidase–linked anti-mouse conjugates (DAKO) according to the standard Western blot protocol. α-Tubulin was included as a loading control. Blots were developed using Supersignal WestPico chemiluminescent substrate (Pierce), imaged and analyzed by the Bio-Rad Gel Imaging System.

### MTT assay

Cells were plated into 96-well flat-bottomed plates (Becton Dickinson, Heidelberg, Germany) at 1.5×10^4^ cells/well in 150 µl culture media, and after 24 or 48 h, the viable cells were assayed for their ability to transform 3-(4, 5-dimethylthiazol-2-yl)-2,5-diphenyltetrazolium bromide (MTT) into purple formazan, and their absorbances at OD490 were determined. All analyses were performed in triplicates.

### Cell cycle analysis

For cell cycle analysis, 48 h after infection or transfection, cells were obtained by trypsinization and pooled with the floating cells and centrifuged at 1000 rpm for 5 min. Propidium iodide (0.05 mg/ml, Sigma) and RNA-seA (0.1 mg/ml, Sigma) were added to the cells and samples were analyzed 30 min after staining with the use of flow cytometry-BD FACSCalibur (BD) and Cell-Quest software.

### Colony formation assay

24 hours after infection or transfection, 50 treated cells were plated into a fresh 24-well plate and maintained in DMEM containing 10% FBS for 2 weeks. Colonies were fixed with methanol and stained with 0.1% crystal violet in 20% methanol for 15 min. Colonies with cell numbers of more than 50 cells per colony were counted. All the experiments were performed in triplicate wells in 3 independent experiments.

### Construction of vectors

As determined by computational predictions, the 3′-UTR of Wnt1 mRNA contains two seed sequences that are partially complementary to miR-152, we thus constructed pGL3-WNT1-1-3′UTR and pGL3-WNT1-2-3′UTR, both of which contain 90 nt (respectively including the seed sequence 1 and seed sequence 2 and some of their flanking sequences). The respective WNT1-1-wt 3′UTR and WNT1-2-wt 3′UTR sequences used were as follows: 5′-CTAGACCCGGACCTACCTCCCTCCCTCTCCGCGGGAGACCCCTTGTTGCACTGCCCCCTGCTTGGCCAGGAGGTGAGAGAAGGATGGGTCCCCTCT-3′ (WNT1-1-wt-F) and 5′-CTAGAGAGGGGACCCATCCTTCTCTCACCTCCTGGCCAAGCAGGGGGCAGTGCAACAAGGGGTCTCCCGCGGAGAGGGAGGGAGGTAGGTCCGGGT-3′ (WNT1-1-wt-R); 5′-CTAGAACCCCTTCCTGTCCTGCCTCCTCATCAGTGTGTAAATAATTTGCACTGAAACGTGGATACAGAGCCACGAGTTTGGATGTTGTAAATAAAT-3′ (WNT1-2-wt-F) and 5′-CTAGATTTATTTACAACATCCAAACTCGTGGCTCTGTATCCACGTTTCAGTGCAAATTATTTACACACTGATGAGGAGGCAGGACAGGAAGGGGTT-3′ (WNT1-2-wt-R); On the other hand, two pGL3 constructs respectively containing the WNT1-1-3′-UTR and WNT1-2-3′-UTR with point mutations in the seed sequence were also constructed, and the WNT1-1-mut 3′UTR and WNT1-2-mut 3′UTR sequence used were respectively shown as follows: 5′-CTAGACCCGGACCTACCTCCCTCCCTCTCCGCGGGAGACCCCTTGTTAGCCCTCCCCCTGCTTGGCCAGGAGGTGAGAGAAGGATGGGTCCCCTCT-3′ (WNT1-1-mut-F) and 5′-CTAGAGAGGGGACCCATCCTTCTCTCACCTCCTGGCCAAGCAGGGGGAGGGCTAACAAGGGGTCTCCCGCGGAGAGGGAGGGAGGTAGGTCCGGGT-3′ (WNT1-1-mut-R); 5′-CTAGAACCCCTTCCTGTCCTGCCTCCTCATCAGTGTGTAAATAATTTAGCCCTAAACGTGGATACAGAGCCACGAGTTTGGATGTTGTAAATAAAT-3′ (WNT1-2-mut-F) and 5′-CTAGATTTATTTACAACATCCAAACTCGTGGCTCTGTATCCACGTTTAGGGCTAAATTATTTACACACTGATGAGGAGGCAGGACAGGAAGGGGTT-3′ (WNT1-2-mut-R).The hsa–miR-152 expression vector pcDNA3.1–hsa–miR-152 contains pri–miR-152 and some of its flanking sequences, and the sequences were cloned into a pcDNA3.1 vector (Promega). All the constructed vectors were confirmed by sequencing.

### Luciferase activity assay

Cells (5×105) were cultured overnight in 6-well plate and co-transfected with 250 ng of pGL3-WNT1-WT or pGL3-WNT1-Mut constructs with 23.5 nM of miR-152 mimics, miR-152 inhibitor, or their negative controls. Each sample was co-transfected with 25 ng of pRL-TK plasmid expressing renilla luciferase to monitor the transfection efficiency (Promega). A luciferase activity assay was performed 48 hours after transfection with the dual luciferase reporter assay system (Promega). The relative luciferase activity was normalized with renilla luciferase activity.

### Statistics

Unless otherwise indicated, the mean and SD were calculated. Between-group comparisons were made with the Mann–Whitney U-test as appropriate. Two-tailed P values<0.05 were considered statistically significant. All statistical analyses were performed with SPSS software (version 17.0).

## Results

### Ad-HCV core infection down-regulated miR-152 in HepG2 cells

**Figure 1:**
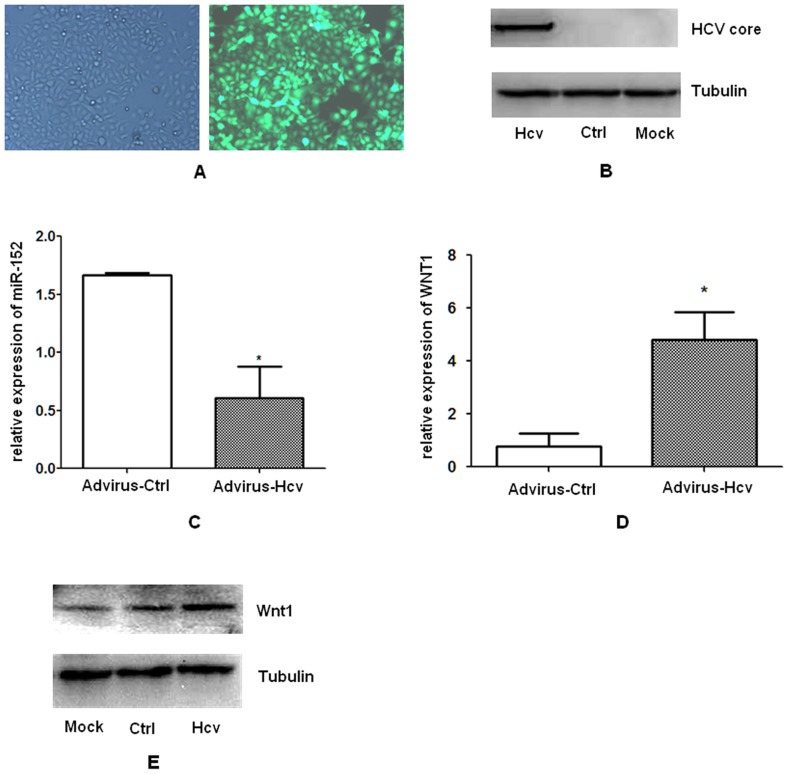
Over-expression of the HCV core protein down-regulated miR-152 expression and up-regulated Wnt1 expression. HepG2 cells were mock infected, or infected with Ad-HCV core or Ad-EGFP for 48 h, and were harvested for preparation of total RNA and protein. (**A**) Infection efficiency of the adenovirus observed under the fluorescent microscope (×10). (**B**) Western blot analysis for HCV core protein expression. (**C**) SLqRT-PCR analysis for miR-152 expression. After normalization to endogenous RNU6B expression, the expression of miR-152 in each group of HepG2 cells was expressed as fold changes in comparison to the levels observed in mock-infected HepG2 cells. (**D**) Real time RT-PCR analysis for Wnt1 mRNA expression. (**E**) Western blot analysis for Wnt1 protein expression. Data are shown as means and standard deviations from triplicate experiments. * P<0.05. (HepG2: mock-infected HepG2 cells; Hcv: HepG2 cells infected with the Ad-HCV core; Co: HepG2 cells infected with the Ad-EGFP control; Advirus-Hcv: Adenovirus expressing the HCV core protein, Ad-HCV core; Advirus-Ctrl: Adenovirus expressing the EGFP control protein, Ad-EGFP).

After confirming potent infection efficacy (Figure 1A) and efficient transient expression of the HCV core protein in HepG2 cells after Ad-HCV core infection at 48 h (Figure 1B), we examined whether miR-152 level was affected by HCV core over-expression. As was shown in Figure 1C, the level of miR-152 was significantly lower in HepG2-HCV core cells than that in the HepG2-EGFP control group.

### Ad-HCV core infection up-regulated Wnt1 expression at both mRNA and protein levels

To investigate whether HCV core protein alters Wnt-1 expression, we measured the levels of Wnt-1 mRNAs and protein after the transient infection of Ad-HCV core into the HepG2 cells. As was shown in Figure 1D and E, HCV core protein induced significant Wnt1 expression at both mRNA and protein level (Figure 1E).

### Ad-HCV core infection promoted cell growth, G1-S transition and colony formation via its down-regulation of miR-152 in HepG2 cells

To test the hypothesis that the HCV core protein would influence the proliferation of HepG2 cells via its down-regulation of miRA-152, we evaluated the effects of transient transfection of miR-152 inhibitor and miR-152 mimics on cell growth using MTT, flow cytometry and colony formation assays.

**Figure 2:**
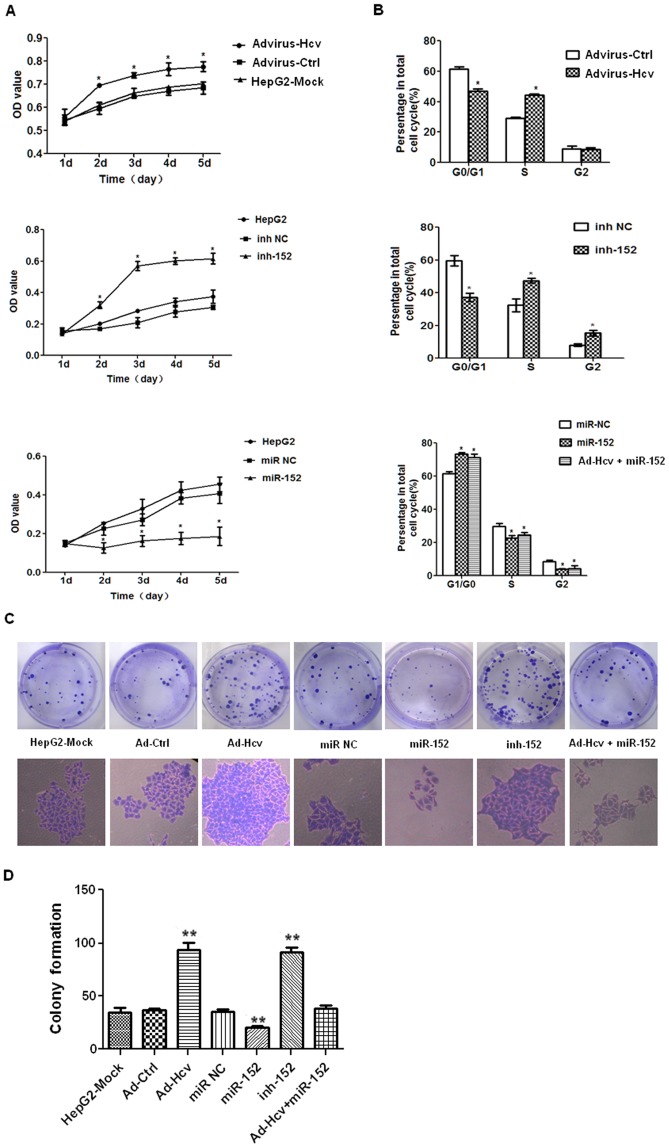
HCV core protein promoted cell growth, G1/S transition and colony formation efficacy of HepG2 cells via its down-regulation on miR-152. HepG2 cells were transfected with double-stranded miR-152 mimics, single-stranded miR-152 inhibitor, or their relative negative control RNA at a final concentration of 5 nM with a HiPerFect Transfection Reagent kit as indicated for 48 h, and were collected for preparation of MTT, flow cytometry and colony formation assay. (**A**) Results of MTT assay. (**B**) Results for cell cycle profiles. (**C**) Results for colony formation assay. Data are shown as means and standard deviations from triplicate experiments. * P<0.05, ** P<0.01. (Advirus-Hcv: Adenovirus expressing the HCV core protein; Advirus-Ctrl: Adenovirus expressing the EGFP control protein; inh NC: scrambled oligonucleotides as the negative control for miR-152 inhibitor; inh-152: miR-152 inhibitor; miR NC: scrambled oligonucleotides as the negative control for miR-152 mimics; miR-152: miR-152 mimics; HCV+ miR-152: Adenovirus expressing the HCV core protein+miR-152 mimics).

We observed profound promotion of cell growth (Figure 2A) by both HCV core protein (upper panel) and miR-152 inhibitor (middle panel), while miR-152 mimics was shown to have significantly inhibited cell growth (Figure 2A, lower panel).

To further investigate the effect of HCV core over-expression on cell cycle, HepG2 cells were infected with Ad-HCV core or Ad-GFP at an MOI = 50, and cells were analyzed for its cell cycle status using flow cytometry after 48 h. As shown in Figure 2B, upper panel, HCV core over-expression induced cell cycle progression from G1 to S phase at 48 h. To test the hypothesis that the HCV core protein promoted G1/S transition via its down-regulation of miRA-152, we performed the gain-of-function and loss-of-function studies of miR-152, and evaluated its effect on cell cycle profiles. We observed profound promotion of G1/S transition by miR-152 inhibitor (Figure 2B, middle panel), while miR-152 mimics was shown to have significantly inhibited G1/S progression (Figure 2B, lower panel).

Colony formation assay showed that the colonies formed by Ad-HCV core infected cells were significantly more (P≤0.001) and larger in size than those of Ad-EGFP infected cells (Figure 2C and D). On the other hand, while the colonies formed by miR-152 mimics-transfected cells were significantly less (P≤0.001) and smaller in size than those of the HepG2-Mock cells, the colonies formed by miR-152 inhibitor-transfected cells were significantly more (P≤0.001) and larger in size than those of the HepG2-Mock cells (Figure 2D).

We further examined the effect of salvage expression of miR-152 on growth characteristics of HepG2-HCV core in flow cytometry and colony formation assays. MiR-152 mimics were transiently transfected into HepG2 cells with previous Ad-HCV core infection for 48 h. Salvage expression of miR-152 in the HepG2-HCV core cells was confirmed by SLqRT-PCR (data not shown). Expectedly, not only was the promoted G1/S transition driven by HCV core over-expression significantly halted (Figure 2B, lower panel), but also was the colony formation capacity substantially inhibited to a basal level (Figure 2C and D), indicating that miR-152 indeed has a growth inhibitory effect and HCV core protein promoted cell cycle progression and colony formation, at least in part, via its down-regulation of miR-152.

### Wnt1 was a target of miR-152

**Figure 3:**
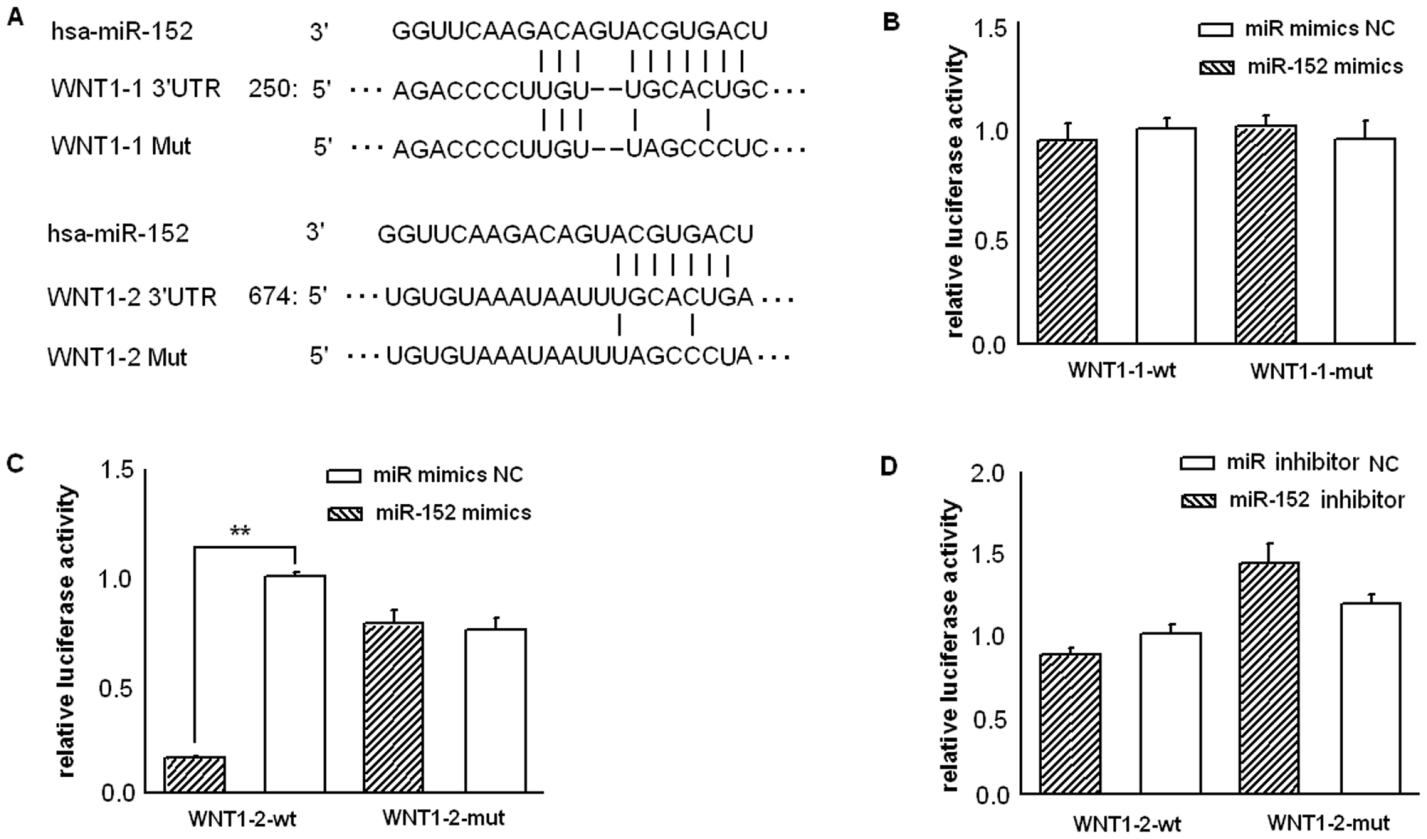
Sequence-specific suppression of Wnt1 gene expression by miR-152. The two WT plasmids, pGL3-WNT1-1-wt-3′UTR and pGL3-WNT1-2-wt-3′UTR, including 90 nt spanning each seed match at the 3′UTR, as well as the two mutant controls, pGL3-WNT1-1-mut-3′UTR and pGL3-WNT1-2-mut-3′UTR, were generated based on the firefly luciferase expressing vector pGL3-promoter and confirmed by sequencing. HepG2 Cells were co-transfected with 250 ng of pGL3-WNT1-WT or pGL3-WNT1-Mut constructs with 23.5 nM of miR-152 mimics, miR-152 inhibitor, or their respective negative control. Each sample was co-transfected with 25 ng of pRL-TK plasmid expressing renilla luciferase to monitor the transfection efficiency. A luciferase activity assay was performed 48 hours after transfection with the dual luciferase reporter assay system (Promega). The relative luciferase activity was normalized with renilla luciferase activity. (**A**) WT and Mut 3′-UTRs of WNT1, indicating the interaction sites between miR-152 and 3′-UTR of WNT1. (**B**) Dual luciferase assay of HepG2 cells co-transfected with the firefly luciferase constructs containing the WNT1-1-WT or WNT1-1-Mut 3′-UTR and miR-152 mimics or its negative control (NC). (**C**) Dual luciferase assay of HepG2 cells co-transfected with the firefly luciferase constructs containing the WNT1-2-WT or WNT1-2-Mut 3′-UTR and miR-152 mimics or miR-152 mimics NC. (**D**) Dual luciferase assay of HepG2 cells co-transfected with the firefly luciferase constructs containing the WNT1-2-WT or WNT1-2-Mut 3′-UTR and miR-152 inhibitor or miR-152 inhibitor NC. Data are shown as means and standard deviations from at least three independent experiments. ** P<0.01.

As predicted by several in silico methods for target gene prediction, including PicTar (http://www.pictar.org/), TargetScan (http://www.targetscan.org), and microRNA (http://www.microrna.org/), the proliferation-associated oncogene implicated in malignant liver cancer growth induced by HCV infection, Wnt1, was identified as one of the high-scoring candidate genes of miR-152 targets, and as was shown in Figure 3A, two miR-152 binding sites (respectively located at 262–268 bp and 688–694 bp 3′-UTR) were indicated at 3′-UTR of the Wnt1 mRNA. To validate the miRNA-target interactions, the Wnt1 complementary sites were cloned into the 3′-UTR of the firefly luciferase gene and co-transfected with miR-152 mimics, miR-152 inhibitor, or their negative contros in HepG2 cells. As shown in Figure 3, while miR-152 mimics didn't reduce the luciferase activity of the WNT1-1-wt-3′-UTR construct (Figure 3B), miR-152 mimics (at a final concentration of 23.5 nM) significantly reduced the luciferase activity of the WNT1-2-wt-3′-UTR construct by 83.76% with respect to its negative control (P<0.0001) (Figure 3C). Conversely, when we performed luciferase assays using a plasmid harboring the mutant 3′-UTR of WNT1-2, the luciferase activity of mutant reporters were not affected by miR-152 mimics (Figure 3C). On the other hand, to explore whether endogenous miR-152 can directly regulate WNT1 via WNT1-2-WT, we tried inhibition of the endogenous miR-152 through the transfection of the miR-152 inhibitor, however, as shown in Figure 3D, miR-152 inhibitor showed no effect on luciferase reporter.

**Figure 4:**
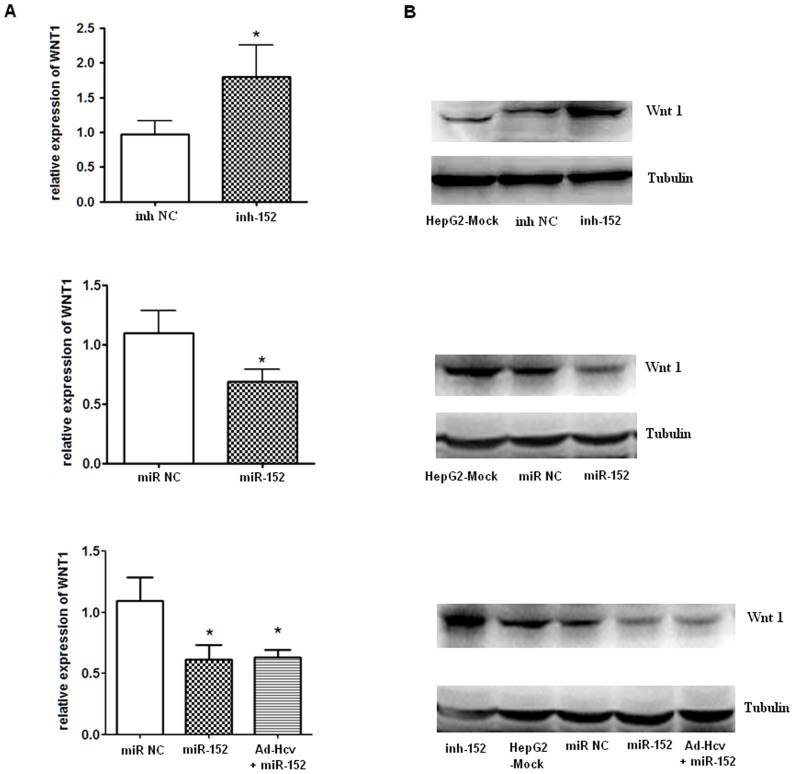
MiR-152 regulates Wnt1 expression at both mRNA and protein level. HepG2 cells were transfected with double-stranded miR-152 mimics, single-stranded miR-152 inhibitor, or their relative negative control RNA at a final concentration of 5 nM with a HiPerFect Transfection Reagent kit as indicated for 48 h, and were collected for preparation of total RNA and protein. (**A**) Real time RT-PCR analysis for Wnt1 mRNA expression. (**B**) Western blot analysis for Wnt1 protein expression. Data are shown as means and standard deviations from triplicate experiments. * P<0.05. (inh NC: scrambled oligonucleotides as the negative control for miR-152 inhibitor; inh-152: miR-152 inhibitor; miR NC: scrambled oligonucleotides as the negative control for miR-152 mimics; miR-152: miR-152 mimics; Hcv+miR-152: Adenovirus expressing the HCV core protein+miR-152 mimics).

To further determine if miR-152 affects Wnt1 expression in the HCC intracellular environment, we analyzed the changes of Wnt1 expression in HepG2 cells after transient transfection of miR-152 mimics or inhibitor. Using real-time-PCR and western blot assays, we found that while miR-152 inhibitor significantly up-regulated Wnt1 expression at both the mRNA (Figure 4A, upper panel) and protein levels (Figure 4B, upper panel), miR-152 mimics dramatically inhibited its expression (Figure 4A and B, middle panel). Most importantly, as was shown in Figure 4A and B, lower panel, salvage expression of miR-152 in HepG2-HCV core cells reversed the increased Wnt1 production mediated by HCV core over-expression, indicating that Ad-HCV core infection might promote cell growth via its down-regulation of miR-152 and the subsequent Wnt1 activation in HepG2 cells.

## Discussion

In the present study, we demonstrated that HCV core protein stimulated proliferation of HepG2 cells with opposite regulations on the expression level of miR-152 and Wnt1. Further studies showed that miR-152 could target the 3′-UTR of Wnt1 mRNA, and suppress its mRNA and protein expression. The inhibition of miR-152 expression by HCV core caused a marked induction of Wnt1 expression. Of particular interest, we showed that salvage expression of miR-152 by miR-152 mimics inhibited the HCV core-promoted production of Wnt1 and cell proliferation. Therefore, miR-152, which is down-regulated by HCV core, can act as a tumor suppressor by inhibiting cell proliferation possibly through regulating Wnt1 in HCC. These results provide functional and mechanistic links between the tumor suppressor miRNA-152 and the oncogene Wnt1 on the proliferative nature of HCV-HCC, highlighting an important role for miR-152 in the regulation of malignant proliferation in the molecular etiology of HCV-related HCC, and suggest a potential application of miR-152 in HCV-related HCC therapy.

The significant growth stimulation (Figure 2A) and reduction of miR-152 expression by the HCV core protein in HepG2 cells (Figure 1), the possible targeting of the proliferation-promoting oncogene, Wnt1, by miR-152 (Figure 3A), and the possible role of Wnt1 over-expression in the mediation of HCV core-induced cell proliferation progression revealed by the work of other groups [24], all prompted us to explore the possible biological significance of miR-152 in cell proliferation in HepG2 cells. After transfection of miR-152 mimics or miR-152 inhibitor into the HepG2 cells for 48 hours, cell cycle profiles were analyzed and colony formation assays were performed. The results revealed that miR-152 inhibitor not only led to obvious G1/S transition (Figure 2B, middle panel), but also promoted colony formation efficacy (Figure 2C, the sixth panel), recapitulating the cell cycle profiles and colony formation-promoting effects mediated by HCV core over-expression in HepG2 cells as was respectively shown in Figure 2B, upper panel and Figure 2C, the third panel. Meanwhile, miR-152 mimics induced a significant G0/G1 phase arrest (Figure 2B, lower panel), and more notably, salvage expression of miR-152 by the transient transfection of miR-152 mimics in HepG2-HCV core cells was show to have significantly inhibited G1/S transition (Figure 2B, lower panel) and colony formation ability (Figure 2C, the seventh panel), indicating the possibility that miR-152 inhibition mediated the cell cycle progression-enhancing effect of HCV core protein.

To test the hypothesis that the HCV core-mediated Wnt1 induction in human liver cells was mediated, at least in part, by the loss of the inhibitory effect of miR-152 on Wnt1, due to reduced miR-152 by HCV core over-expression, we subsequently assessed Wnt1 as a potential functional target of miR-152. Our results showed that miR-152 bound to the complementary sites of 3′-UTR of Wnt1 (Figure 3C), and dramatically decreased its expression at both mRNA and protein levels (Figure 4). More notably, salvage expression of miR-152 by the transient transfection of miR-152 mimics in HepG2-HCV core cells was show to have significantly inhibited Wnt1 expression (Figure 4A and B, lower panels) to an extent similar to miR-152 mimics-treated HepG2 cells, indicating that HCV core-induced Wnt1 induction in HepG2 cells was mediated, at least in part, by the loss of the inhibitory effect of miR-152 on Wnt1. To our knowledge, these observations provide the first line of evidence that miR-152 mechanistically acts via its regulation on Wnt1.

In our previous study, the full 3′-UTR of the human WNT1 mRNA (Genbank accession no. NM_005430) was amplified and cloned into the vector pGL3-promoter, and only minor effects of the miR-152 mimics were shown on the reporter. Given the minor effects of the miR-152 mimics on the reporter in the previous experimental system and the possible interference from the remaining sequence (excluding the seed sequence), we further constructed pGL3-WNT1-1-3′UTR and pGL3-WNT1-2-3′UTR, both of which only contain 90 nt (respectively including the seed sequence 1 and seed sequence 2 and some of their flanking sequences), results showed that while the WNT1-1-wt-3′-UTR site is not regulated by miR-152, miR-152 mimics significantly reduced the luciferase activity of the WNT1-2-wt-3′-UTR construct, suggesting that other sequences in the 3′UTR of WNT1 may have reduced the repression of miR-152 on WNT1. In order to demonstrate that endogenous miR-152 can directly regulate WNT1 via WNT1-2-WT, it is necessary to show relief of luciferase repression from the pGL3-WNT1-2 by inhibiting endogenous miR-152. Therefore, we tried inhibition of the endogenous miR-152 through the transfection of the miR-152 inhibitor, however, we witnessed no effect of miR-152 inhibitor on the WNT1-2-WT luciferase reporter (Figure 3D). Furthermore, the fact that the luciferase expression from the WNT1-2-mut reporter is not higher than from the WNT1-2-wt reporter (Figure 3C) also implies that endogenous miR-152 does not regulate Wnt1 3′UTR. Hence, the observed effects of endogenous miR-152 on Wnt1 expression may be indirect. Our result that miR-152 over-expression repressed luciferase production via WNT1-2-WT demonstrates that modulation of Wnt1 by miR-152 can occur, but possibly only at concentrations of the miRNA that are not achieved in the HepG2 cells.

It has been reported that Wnt1 is up-regulated in several types of human cancer, including HCV-related HCC [24], [25]. More notably, up-regulation of Wnt-1 expression has been shown to positively correlate with tumor proliferation stimulated by HCV core protein [24], and recently, its prognostic role in HCV-related HCC has been demonstrated [26], which is consistent with our findings that down-regulation of miR-152 is associated with a more proliferative HCC phenotype. It does appear, therefore, that in our HepG2 cells, miR-152 modulates proliferation via regulation of Wnt1.

The mechanism by which HCV core regulates transcription has been proposed to be indirect, with the core protein interacting with cytoplasmic signal-transduction molecules and leading to modulation of transcription for genes dependent on these cascades. previous reports have implicated HCV core in the activation of the Wnt/β-catenin pathway. Indeed, expression of HCV core protein has been shown to induce cell proliferation, DNA synthesis, and cell-cycle progression either alone or in the context of HCV replication, which is mediated by transcriptional upregulation of growth-related genes, in particular wnt-1 and its downstream target gene wisp-2 [24]. In this study, we further observed that salvage expression of miR-152 reversed the enhanced G1/S transition, colony formation ability and Wnt1 production mediated by HCV core over-expression; meanwhile, knockdown of miR-152 expression in HepG2 cells substantially promoted cell proliferation and Wnt1 production to an extent similar to that in HepG2-HCV core cells. These results support our theory that miR-152 might be a predominant mediator of HCV core-promoted Wnt1 production and proliferation in HCC cells, suggesting that over-expression of HCV core may result in a down-regulated expression of miR-152 and, subsequently, the induction of Wnt1 and cell proliferation.

In summary, in this study, we investigated the potential role of miR-152 in HCV core-mediated HCC proliferation and its underlying mechanisms. Our data suggested that HCV core-promoted down-regulation of miR-152 played an important role in HCC cell proliferation, possibly through the indirect targeting of Wnt1 by miR-152. The identification of the direct target of miR-152, which in turn leads to the regulation of Wnt1, is still underway. MiR-152 could be employed as an effective therapeutic target for HCV-HCC.
